# Scrutinizing microbiome determinism: why deterministic hypotheses about the microbiome are conceptually ungrounded

**DOI:** 10.1007/s40656-024-00610-0

**Published:** 2024-02-12

**Authors:** Javier Suárez

**Affiliations:** 1 BIOETHICS Research Group - Department of Philosophy, University of Oviedo, Oviedo, Spain; 2 Institute of Philosophy, Jagiellonian University, Kraków, Poland

**Keywords:** Postgenomic determinism, Microbiota, Microbiome heritability, Phylosymbiosis, Hologenome, Causality

## Abstract

This paper addresses the topic of determinism in contemporary microbiome research. I distinguish two types of deterministic claims about the microbiome, and I show evidence that both types of claims are present in the contemporary literature. First, the idea that the host genetics determines the composition of the microbiome which I call “host-microbiome determinism”. Second, the idea that the genetics of the holobiont (the individual unit composed by a host plus its microbiome) determines the expression of certain phenotypic traits, which I call “microbiome-phenotype determinism”. Drawing on the stability of traits conception of individuality (Suárez in Hist Philos Life Sci 42:11, 2020) I argue that none of these deterministic hypotheses is grounded on our current knowledge of how the holobiont is transgenerationally assembled, nor how it expresses its phenotypic traits.

## Introduction

The completion of the Human Genome Project in the early 2000s strongly suggested that, even if the genomic content of an organism is relevant to know some of its phenotypic features, it is by no means the “holy grail” that would allow uncovering all the determinants of the human phenotype—including the determinants of human behaviour—as some had incorrectly supposed. As a matter of fact, instead of revealing the genetic determinants of the human phenotype, the Human Genome Project revealed that the phenotype is a polyhedral product of multiple and heterogeneous factors, including both nature (genetic determinants) and nurture (developmental and environmental cues). This influenced the emergence of the so-called “post-genomic” approaches to the study of life, in which the number and kind of molecular elements affecting the phenotype of the organism expands over and above its genome (Griffiths & Stotz, 2013). This movement apparently puts organisms and their causal complexity at the centre of the debate, partially involving a movement from the century of the gene—as Fox Keller (2000) baptized the twentieth century—to the century of the organism (Baedke, 2019; Montévil et al., 2018). Post-genomic approaches to the study of life include the study of epigenetic factors, the interaction between the genotype and the environment (e.g., niche construction theory), a closer look to the patterns of gene expression (functional genomics, proteomics), or the study of the microbiome, among others. The beginning of the postgenomic era suggested that some of the topics and controversial philosophical assumptions embedded in the Human Genome Project could be overcome, including its tendency towards essentialising the human nature, its lineal conception of causality (from genes, to proteins, and from proteins to phenotypes), its genocentrism and genoreductionism, and its biases towards genetic determinism (Gannett, 2019).^1^

However, in recent years, many have emphasised that the postgenomic era is ultimately grounded in the same (or some of the same) underlying philosophical assumptions that grounded the Human Genome Project. For example, in a rich and fundamental work on the topic, Meloni & Testa (2014, p. 439, original emphasis) show that a paradox in postgenomic research “rest(s) on [its] bivalent [and, I’d say, contradictory] understanding of its relationship with genomics: on the one hand *as a missing link that can succeed where genomics purportedly failed*, on the other *as a quantum leap enabled by the very success of genomics*”. Note that the first reading entails that postgenomic research inherits all the problematic philosophical assumptions embedded in the Human Genome Project, as the very conceptual or ontological foundations would remain unaltered. In a similar vein, Waggoner & Uller (2015) argue, based on the type of language used in at least three areas of postgenomic research (“genetic” control, programming, and inheritance), that postgenomic approaches restate the determinism but in a form of environmental determinism. To quote: “[Postgenomic language] may simultaneously promote a novel form of determinism, one that highlights the “influential” role of the environment and behaviour in *determining* individual characteristics and even the expressed genetic code of future generations” (Waggoner & Uller, 2015. p. 178, emphasis added). Similar claims can be found in more recent works, such as Richardson & Stevens (2015), Dubois et al. (2018), Santaló (2018) or Dupras et al. (2019). An area of particular interest in this context is microbiome research, which also put forward its own version of the Human Genome Project by launching the Human Microbiome Project in 2008 (Turnbaugh et al., 2007; see https://www.hmpdacc.org/ for all the information about the Human Microbiome Project between 2008 and 2016; web last accessed on 28/12/2023). My goal in this paper is to examine how microbiome research implicitly endorses deterministic conceptions on its own, what these conceptions specifically consist in, what they are grounded on, and their limitations given our contemporary knowledge of the microbiome and its relationship to host biology.

Before proceeding, I would like to clarify that this paper is not about the potential causal role of the microbiome, which has been abundantly discussed in the literature so far (Hanage, 2014; Cani, 2018; Bourrat, 2018; Walter et al., 2020; Lynch et al., 2019; O’Malley & Parke, 2020), but rather about a specific dimension of this causal role. While the topic of determinism and the topic of causality may be linked, they are conceptually different (Hoefer, 2016). In biology, genetic—and postgenomic, as I will show—determinism concerns whether the genetic fixes the traits that an organism expresses. In this sense, genetic determinism presupposes that causation works bottom-up (from genes to phenotypic traits) and that the genes play the most important causal role in fixing the traits. In contrast with this, the debate about causality admits different degrees or forms in which causal claims can be made, and includes different types of causal influences or interactions, including both top-down and bottom-up forms. To put it differently, the issue of determinism concerns whether the strength of the nomic relationship between a specific cause and an effect is enough for the cause to be determining that the effect is primarily produced by such cause. Thus, even if my conclusions will suggest that deterministic claims about the microbiome—in the two forms I will distinguish later—are misguided, the reader should not presuppose that I am simultaneously arguing the microbiome does not play any type of causal role for its host.^2^

My agenda will be as follows. In Sect. 2, I introduce the concept of genetic determinism as it is used in today’s biological research. In Sect. 3 I motivate the application of the notion to microbiome science by distinguishing between “host-microbiome determinism” and “microbiome-phenotype determinism”. In the second part of this section, I offer an analysis of the most common methodological practices used and the main question raised in today’s microbiome research to illustrate the specific character of these two types of determinism. In Sect. 4, by drawing on our current knowledge of host-microbiome systems, I show that both types of determinism are ungrounded. Finally, in Sect. 5, I present my conclusions.

## Genetic determinism

Roughly speaking, genetic or biological determinism is the belief that the traits expressed by an organism during its life cycle are a product of its genetic composition. These traits range from basic phenotypic traits such as eye or skin colour, the capacity for tongue rolling, or boldness, to more complex traits such as cancer, schizophrenia, diabetes, or Alzheimer’s disease, and also the so-called social or personality traits like openness, extraversion or sexuality phenotypes such as being gay. Genetic determinism is usually motivated by the correct observation that the expression of some traits is strongly connected to the presence or absence of specific genes or alleles (Sarkar, 1998, p. 10). For example, if a person inherits two mutations (one maternal, one paternal) in the beta globin gene, she will likely develop sickle cell anaemia; and if a person inherits a trisomy in the chromosome number 21, she will likely be born with Down syndrome. A key potential consequence of this basic and under elaborated view of genetic determinism is thus that environmental interventions are useless when it comes to modify the expression of certain traits, because their expression depends on the genetic composition of the organism expressing the traits. In other words, if a trait is genetically determined, then the organism bearing the genes *for* the trait will carry the trait, no matter how many efforts one makes in altering the environment to avoid its instantiation (Rosoff & Rosenberg, 2006, p. 123).

When it comes to genetic determinism, however, it is convenient to distinguish different meanings, as the expression is polysemic, and it is not always clear what concept of genetic determinism is being used across different contexts (Gayon, 2009; Kaplan, 2000). Part of the problem stands from the complexity of the concept of determinism itself, and its relationship to debates about human agency, causality of the future, and prediction (Hoefer, 2016; Müller & Placek, 2018). In fact, some of the arguments *against* genetic determinism emphasise the complexity and the lack of predictability of some biological facts (Robertoux & Carlier, 2011), whereas others put their emphasis on the plurality of (causal) factors beyond genetics affecting human nature (Dupré, 2003). These two extreme visions of what genetic determinism is, and the responses that need to be articulated to resist genetic deterministic claims, reflect the existence of at least two different concepts potentially meant by the expression. On the one hand, what Kaplan (2000) calls the “complete information” strand, and Gayon (2009) names “Laplacian (scientific) determinism”. This is an epistemological interpretation of genetic determinism, according to which the genetics of an organism provide complete information about its phenotype, to the point that if the details of the genotype were fully known, it would be possible to predict the complete phenotype that the organism would develop during its ontogeny. Kaplan (2000, p. 11) argues that this form of determinism is considered *trivially* false, although as a matter of fact some researchers defend its falsehood on the basis of the complex mapping that exists between the genotype of an organism and its phenotype (Roubertoux & Carlier, 2011).

On the other hand, what Kaplan (2000) names the “intervention is useless” strand, which can roughly be equated to Gayon’s (2009) “Laplacian (metaphysical/ontological) determinism”. The main idea underlying this interpretation is that the genes fix the phenotypic traits to the point that the possession of one gene determines the expression of the trait, regardless of the potential interventions in the environment. To give an example, if sickle cell anaemia is fixed by having two mutations in the beta globin gene, then it does not matter how we modify the environment of a person who carries two copies of the gene, because she will bear the trait. Note that this conception of genetic determinism is not strictly the same as the type of determinism used in physics or in other disciplines. The point here is not that the genes *strictly fix* the phenotype, but rather that *they constrain* its expression so narrowly that human intervention in the environment is unwarranted. “Intervention is useless” genetic determinism is also almost impossible to maintain, both in its application to simple traits—with phenylketonuria as a classic example of why this conception of genetic determinism does not hold—but particularly in its application to complex diseases as well as social or personality traits, where the potential for intervention is especially broad, and biologists are generally conscious of this (Ellison & De Wet, 2018).

Given that these two conceptions of genetic determinism are inadequate to capture what biologists mean when they use the expression, as well as to capture the research founded on deterministic hypotheses that is carried out in today’s biology, Kaplan urges to consider a third meaning that he characterizes as the conjunction of two theses, one methodological and the other ontological: “(a) the genetic is the natural place to look when attempting to explain, predict, and control traits with even partial genetic etiologies, and (b) traits with partial genetic etiologies are best understood as being *primarily* genetic, and it is only through *directed* intervention that the expression of genes for traits with partial genetic etiologies can be avoided or controlled” (Kaplan, 2000, p. 12). In this context, *genetic* means something similar to “vertically inherited”, i.e., passed from parent to offspring during zygote formation (cf. Merlin & Roboli-Sasco, 2021; Veigl et al., 2022). Under this conception, genetic determinism is a driving hypothesis of biological research which encourages researchers to look for the hereditary basis of the trait (methodological primacy of the genetic) as it is assumed that its expression can be manipulated by controlling such basis (ontological control by the genetic). In a motto, this concept of genetic determinism can be summarized in the hope of “controlling the trait by controlling the genetic”. Kaplan argues that this third form of genetic determinism is present in most of today’s biological research, and will usually explicitly appear due to the emphasis of the power of the genetic in allowing both predictions and control.

It must be noted at this point how the issues of genetic determinism and biological causality differ. Claims about biological causality may emphasise that a specific factor plays a causal role in producing a trait, for example, if someone appeals to the joint role of developmental constraints and selective effects in producing a specific form of a trait, like the obstetric conundrum (Grunstra et al., 2019). In these cases, while biologists clearly make causal claims, some of which involve the genetic level, it makes little sense to speak of genetic determinism, because the genetic is at the same level as other factors, and the evolution of the trait is considered complex, instable, and highly contingent. Thus, genetic determinism differs from genetic causality in that the former, but not the latter, prioritizes the genetic over any other factor. Secondly, biological claims about causality may be both top-down or downward and bottom-up or upward (see Sect. 4). For instance, research on natural selection may appeal to how selection on traits expressed at the higher-level causes the distribution of genetic forms in the lower-level to differ from what would be expected if natural selection was a cause of change by acting exclusively on the lower-level (Lloyd & Wade 2019; Suárez & Triviño, 2019; Suárez & Lloyd, 2023). When causation is predicated downwardly, it makes little sense to talk of genetic determinism, as one would be presupposing that the genetic is neither methodologically nor ontologically privileged, but just an effect of what happens in non-genetic levels (organism-environment interaction). However, this would be a case of biological causality. Therefore, biological determinism, as it is used in today’s biology, concerns both the strength and the direction of the causation, whereas the range of potential causal claims being made in biology is wider. Grounded on this conception, in the next section, I show how microbiome research introduces deterministic considerations on its own, and how they relate to Kaplan’s concept of genetic determinism.

## Two types of microbiome determinism: host-microbiome determinism and microbiome-phenotype determinism

Microbiome research is a growing field in contemporary biology which studies the microbial composition of multicellular organisms, specially animals and plants, as well as their influence on animal phenotype, including behaviour, physiology, reproduction and health. The growth of microbiome research started in early 2000, with the development of high-throughput sequencing technologies. These technologies allowed divorcing bacterial identification from bacterial culture, and permitted discovering that animal and plants harboured millions of microbes in their bodies. This led some authors to interpret that this discovery should have profound implications for our understanding of biological individuality (Gilbert et al., 2012; McFall-Ngai et al., 2013; Stencel & Proszewska, 2017; Suárez, 2018). For example, Dupré (2010) argued that organisms should now be conceived as polygenomic (de-essentialized) entities, whose expressed traits were highly indetermistic as they depended on the unfolding of several genomes (see also Dupré, 2021). However, contrary to the expectations, it led some to consider that the microbiome constitutes “a second genome”, serving as a source of genetic variation that could even hypothetically account for phenomena like the missing heritability problem (Sandoval-Motta et al., 2017). This led to the introduction of deterministic conceptions in microbiome research, conceived in terms Kaplan’s third meaning of “determinism” (Sect. 2). While he introduced this third meaning for studies carried out under the hopes raised by the Human Genome Project, I think the conception of genetic determinism he offers can serve as a basis to understand the very specific forms of determinism that are implicit in today’s research on the microbiome, even if the latter supposes an expansion of the former as I will show later. Particularly, in most microbiome research it is also usually assumed that the genetic determines the expression of certain traits: (a) by being a methodologically privileged place to investigate the source of these traits and (b) by playing a primary causal role in their expression, provided these traits have partial genetic aetiologies.

The analogy between genetic and microbiome determinism is grounded on the observation that the microbiome affects the expression of several host traits. Well-described cases include: the influence of *Vibrio fischeri* on the bioluminiscense of the Hawaiian bobtail squid; the role of different *Treponema* species in favouring the production of short-chain fatty acids from wood fibres in *Nasutitermes takasagoensis*; the role of cellular microbes of the exoskeleton of the leaf-cutting ant *Acromyrmex echinatior* in nestmate recognition; or the role of *Bacteroides plebeius* in the digestion of seaweed in humans (see Lynch & Hsiao, 2019 for a review of these and other examples). While this evidence opens up the possibility of thinking that host traits can eventually be primarily modified by manipulating the microbiome, the situation is not as simple, for the microbiome needs to be assembled de novo each generation (Chiu & Gilbert, 2015). Furthermore, most of the microbes composing the microbiome of a host are acquired horizontally, during the life cycle of the organism. While traces of vertical transmission can be found for some microbial species and some animal taxa, they constitute a minority. The host has thus to assemble its microbiome “afresh” each generation. Therefore, there is no a priori guarantee that the microbial components being acquired belong to the same lineage, or even to the same taxa, as the components borne by their progenitors. The key question that microbiome researchers need to ask before considering whether the microbiome can play a primary causal role in determining the host phenotype is whether there is any host determinant conditioning microbiome assembly, or rather the process that depends on other factors (e.g., ecological or behavioural). In most microbiome research, it is further assumed that the primary host determinant of microbiome assembly must be the host genotype, and that the components to be acquired correspond to the genetic components of the microbiome. It is thus inferred that if the microbiome were reassembled by factors that do not depend on the genotype of the host, this would hamper the potential for “controlling the trait by controlling the genetic”, where *genetic* refers to the genetic composition of the microbiome. This assumption grounds that many deterministic practices from the Human Genome Projects have been extended to microbiome research, and this extension came hand in hand with an extension and refinement of the older deterministic views.

Henceforth, an important part of microbiome research is aimed at showing: On the one hand, that microbiome assembly is not primarily guided by ecological or behavioural processes, but it is rather strongly determined by the host genotype. This type of research gives rise to what I call “host-microbiome determinism”; on the other hand, that the microbiome influences on part of the host phenotype in a substantial way, having causal primacy over other factors for the production of certain traits. This gives rise to what I call “microbiome-phenotype determinism”.^3^ Both forms of determinism are specifications of Kaplan’s third meaning of genetic determinism, as these are applied in microbiome research. This occurs because both forms of microbiome determinism entail an excessive reliance on the genetic and the molecular as *the primary* causal agents of some traits, and in doing so they entail that other causal factors (some of which may ultimately play a more prominent causal role than the genetic in the production of the trait) are ignored. This a crucial feature of the denounce of microbiome determinism that I am making. Table 1 introduces a comparison of the two types of determinism isolated in this work.^4^

**Table 1:** Two types of determinism in microbiome research

Form of Determinism	Host-microbiome determinism	Microbiome-phenotype determinism
Type of Thesis	*Host genetics → Microbiome composition*	*Microbiome composition → Host Phenotype*
Methodological Thesis	The host genetics is the most adequate place to investigate, predict or explain the hereditary basis of the microbiome	The microbiome is the most adequate place to investigate, predict or explain these host traits with partial microbiome aetiologies
Ontological Thesis	Microbiome composition is primarily controlled by the host genetics	Certain host traits are under the control of the microbiome primarily
Underlying deterministic assumptions	The microbiome is causally assembled by the host genetics Microbiome assembly is due to the host genotype	The effects of the microbiome on those host traits that are partially induced by the microbiome have causal primacy over other factors
	*Consequence*: Non-genetic (ecological, behavioural, etc.) factors play a minor role and can be ignored	*Consequence*: Non-microbiome factors play a minor role and can be ignored

I contend that these two lines of research in contemporary microbiome science constitute two ways in which the old non-trivial deterministic inclinations embedded in the Human Genome Project and diagnosed by Kaplan re-appear in the context of microbiome science. In other words, I claim that the hopes of “controlling the trait by controlling the genetic” and, in doing so, masking the study of non-genetic factors influencing the expression of the phenotypic traits, are still fully present in some parts of microbiome science, and they manifest specifically in “host-microbiome determinism” and “microbiome-phenotype determinism”. In what follows, I explain in detail what these two form of determinism consist in, show how they manifest in today’s microbiome research and the dangers they entail.

### Host-microbiome determinism

Host-microbiome determinism focuses on the host genetics to predict and eventually control the composition of the microbiome. The guiding assumption is that microbiome assembly is largely determined by the host genetics, thus the relevance of other potential causal factors being diminished. The microbiome (understood as the sum of genetic sequences of microbial taxa identified) is conceived as a trait of the host and, as such, it must be possible to uncover the genetic differences that account for the intrapopulational variability in microbiome composition. In this vein, the driving deterministic theses assumed in host-microbiome determinism are: (a) that the host genetics is the most adequate place to investigate the hereditary basis of the microbiome (methodological thesis); (b) that the microbiome composition could be primarily manipulated by controlling the host genetics, with the latter acquiring primacy over controlling other potential causal factors such as diet or seasonality (ontological thesis).

The main source for host-microbiome determinism in today’s microbiome research is primarily found in twin microbiome studies (TMS, hereafter), and on microbiome genome-wide association studies (mGWAS, hereafter), each based on traditional genetic twin studies and genome-wide association studies (GWAS, hereafter) respectively. Twin studies have traditionally been the main source for determining heritability estimates. They focus on the phenotypic variation between two identical twins to calculate the fraction of phenotypic variation with respect to a trait that may be due to genetic differences, and the fraction that may be due to environmental variables. As identical twins are supposed to share their genetic basis, all the realized phenotypic variation found among them is attributed to the environment. Twin studies face several challenges, including the difficulty of finding twins, problems of randomization or the difficulty for estimation that the phenotypic variation being found is reliably connected to the environment, given identical twins tend to share the same environment (Sahu & Prasuna, 2016). In contrast, GWAS do not require twins, as they concentrate on big cohorts of individuals divided in two groups: a group bearing the traits of interest (e.g., having a specific disease) and another lacking the trait. In these studies, the whole genome is sequenced in the hope of finding some genetic differences between the group bearing the trait and the group lacking the trait. GWAS are usually oriented towards finding single nucleotide polymorphism (SNP, hereafter), that could account for the differences in the trait. GWAS also face several challenges: first, they do not report causality, but at most describe how certain differences correlate to each other; second, they cannot find epistasis, which is known to be an important driver of phenotypic expression; third, they cannot account for population stratification, which is in many cases causally connected to genetic differences within a population but disconnected from some of the phenotypic differences of interest in GWAS (Tam et al., 2019).

In the case of the microbiome, like traditional twin studies, TMS consider the microbiome to be a trait of the host and investigate whether identical twins have a similar microbiome composition or species abundance in their microbiomes. The driving hypothesis is that part of the diversity in microbiome composition derives from diversity in host genetics. As such the microbiome should be more similar in composition and abundance in identical twins than in non-identical twins, and it should also be more similar between identical or non-identical twins than between these and any other member of the population. TMS frequently rely on 16S rRNA analysis as the primary sequencing technology for determining species diversity (Boem & Suárez 2024). 16S rRNA analysis consists in sequencing only the 16S subunit (1542 nucleotides) of the ribosomal RNA, which is a well-known and (almost) universal structural gene in bacteria, commonly used as marker of phylogeny. The use of 16S rRNA sequencing contrasts with the possibility of using alternative technologies that would provide more information, but are more expensive, and harder to use (Suárez & Boem, 2022). Important TMS include Falony et al. (2016), Goodrich et al. (2014a) Goodrich et al. (2014b), Le Roy et al. (2018), Tims et al. (2013), Turnbaugh et al. (2009), Weissbrod et al. (2018), Yatsunenko et al. (2012), Xie et al. (2016).

mGWAS studies, in contrast with twin studies, seek to find the correlation between genetic variants in a human population, and genetic variability in the microbiome composition and/or abundance in the same population. Normally, but not always, mGWAS studies seek a triple association, between SNP, microbiome differences and disease states, suggesting that in these studies host-microbiome determinism is combined with microbiome-phenotype determinism (see below). mGWAS studies frequently rely on 16S rRNA analysis of the microbiome, which limits the information they provide, although shotgun metagenomics sequencing is becoming more frequent (Boem & Suárez, 2024). Important mGWAS studies include Blekhman et al. (2018), Bonder et al. (2016), Davenport et al. (2015), Goodrich et al. (2016), Hua et al. (2016), Igartua et al. (2017), Rothschild et al. (2018), Rühlemann et al. (2018), Turpin et al. (2016), Wang et al. (2016).

But how and why TMS and mGWAS studies presume deterministic hypotheses about the host-microbiome association? Recall that host-microbiome determinism consisted of both a methodological and an ontological thesis, both supported by the underlying assumption that the host genetics was the primary agent causing microbiome assembly, with the latter understood as the sum of genetic sequences of microbial taxa identified and assumed to be a host trait (Table 1). Methodologically, researchers relying on the use of TMS and mGWAS assume that the host genetics is the appropriate place to investigate, predict or explain the so-called trait of microbiome assembly. But note that the sole insistence in studying the priority role of the host genetics in microbiome assembly, and reducing the latter to a host trait, is a subtle form of determinism, analogous to the type of sophisticated determinisms carefully isolated by Kaplan within the context of the Human Genome Project. By reducing microbiome assembly to a host trait potentially under the control of the genetics of the host, TMS and mGWAS studies are therefore methodologically (and problematically) prioritizing the investigation of the genetic side of host-microbiome assembly, excluding the study of other important determinants such as the ecology, behaviour or immunology of the host, as well as the ecological dynamics of the microbiome. Host-microbiome methodological determinism thus appears because the reliance on methodologies such as TMS and mGWAS displaces the investigation of other potential sources of microbiome assembly. For example, Rühlemann et al.’s (2018) mGWAS analysis is explicitly described as adopting a holistic approach, in treating the host, its genetics and its microbiome as a single “metaorganism”. However, the analysis fails to be really holistic, for it only seeks to find a correlation between certain host genetic loci and microbiome composition. The assumption of the study was hence that the presence of the specific genetic loci would determine, in a priviledged way, the microbiome composition. But network effects of how different microbial species ecologically interact with one another and how this ultimately affects microbiome composition were ignored. Similarly, Goodrich et al. (2016) TMS simply showed how certain host genes were associated with the abundance of certain bacterial taxa. While the study is interesting and useful in measuring heritability, it masks potential network effects between the microbes composing the microbiome of their sample population. Note that I am not claiming that microbiome scientists should not investigate whether the genetics of the host may have some influence in microbiome assembly (as Kaplan is not denying that the genetic must be studied). I am only saying that: (1) this type of studies require the consideration of the microbiome and its assembly as a host trait; (2) such consideration is linked to the methodological assumption of a role of the genetics of the host in determining it; (3) this type of research problematically prevents studying other potential sources of microbiome assembly which must accompany these type of studies to avoid the risk of repeating the fallacies of other old forms of determinism.

Ontological host-microbiome determinism, in contrast, derives from the assumption about the causal primacy of the host genetics in determining microbiome assembly. The position is manifested in two facts. First, that a positive result (e.g., a clear observation that a difference in microbiome composition is correlated with a difference in the host genome composition) frequently leads to the proposition that microbiome assembly is not random, but rather influenced by the host genetics. But, unfortunately, other potential options leading to the same or strikingly similar microbiome assemblies are not investigated. This problem is especially acute in the case of mGWAS, for the fact that two genetic variants within a population assemble their microbiome differently may be a result of a common cause, for example, that the genetic differences determine distinct behaviours or nutrition patterns which, ultimately, lead to divergent microbiome compositions (Vuong et al., 2017; Nagpal & Cryan, 2021). Second, the fact that the contrasting explanatory class for the cases where the host genetics cannot explain microbiome divergence are characterized as cases where the assembly is “random” or “stochastic”. For instance, in a recent study, Dove et al. (2021) analyse the drivers of microbiome assembly, and classify them between factors driven due to selection (i.e., those due to the host genetics and a history of positive selection) and factors driven by stochasticity. In another study, Furman et al. (2020) distinguish between deterministic and stochastic drivers. Host diet and age would be deterministic drivers, whereas host ecological encounters in early life would be stochastic events. Interestingly, host diet and age can be ultimately linked to host genetics, whereas the encounters of the host in early life cannot. Note that the classification in the factors driving microbiome assembly into deterministic and stochastic only makes sense provided all the potential causal determinants of microbiome composition that go beyond the genetics of the host are taken to be non-causal (or non-causal enough for the purposes of microbiome scientists). This is unfortunately a mistake, one which specifically ignores the complex biology of host-microbiome systems, including the ways in which the microbiome is transgenerationally assembled, as I will show in Sect. 4.

Note that neither TMS, nor mGWAS are exclusive to humans. Many of these studies are also carried out with animals (Rawls et al., 2006; Blekhman et al., 2018; Wang et al., 2016). This suggests that host-microbiome determinism is possibly widespread and affects biology as a whole, and not biomedicine exclusively.

### Microbiome-phenotype determinism

Microbiome-phenotype determinism operates by assuming that the host and the microbiome must be considered as an entity that expresses a unique phenotype in interaction. While this thesis does not need to be a priori deterministic (see Sect. 4), it is frequently accompanied by the hope (and hype) that control over the microbiome would provide control over the host phenotype for these traits with even partial microbiome aetiologies, and that this type of control should be prioritized over other potential causal factors due to its potential. This expectancy is often grounded on the belief that the microbiome is partially hereditary or, at least, that the subset of the microbiome directly affecting the trait expression is transgenerationally passed from parent to offspring. Sometimes, this is due to the fact that the research concentrates exclusively on one or a few symbionts known to be transmitted vertically. At other times it focuses on microbes that are environmentally abundant, and thus it is almost guaranteed that they will be part of microbiome assembly. In any case, this type of determinism presupposes that the microbiome has causal primacy in the production of some phenotypic effects of its host. It follows from this that microbiome-phenotype determinism assumes: (a) that the microbiome is the most adequate place to predict or explain these host traits with partial microbiome aetiologies; (b) that certain host traits are under the control of the microbiome.

Microbiome-phenotype determinism is becoming increasingly popular in today’s biological research. Most of the studies drawing on this type of determinism presuppose that the microbiome itself controls, regulates, or determines certain expressed phenotypic traits (ontological microbiome-phenotype determinism). The driving idea is that the microbiome constitutes a pool of genetic variation for the host which ultimately affects in a primary manner some of the traits that the latter expresses. While today’s biological evidence undoubtedly suggests that there is a causal connection between the microbiome and host traits, microbiome-phenotype determinism is not just a thesis about causal influence, but rather about causal *primacy*. Namely, it is the thesis that the microbiome has a *primacy* in the expression of these traits. For example, several studies on how the microbiome determines certain host diseases focus on how some bacterial species are present or absent in patients with a specific disease, under the assumption that presence or absence would primarily cause the disease. In other occasions, studies reveal changes in the relative densities of specific species, with some increasing and others decreasing (see Madhogaria et al., 2022 for a review of several studies). While both methods can be important for detecting what is happening, it is surprising that none of these studies focus on the ecological complexity driving such changes. Instead, they focus solely on the very changes themselves (what new species there are, how many new members are present). This reveals a deterministic assumption about the components primarily causing the state, rather than how these components are ecologically arranged. Additionally, research drawing on this form of determinism usually also presupposes that the microbiome, conceived exclusively in genetic terms, is the best place to look when trying to investigate, predict or explain certain host traits with partial microbiome aetiologies (methodological microbiome-phenotype determinism). While this thesis seems to depend on the former, as the prediction or explanation is supposed to be valid *because* the microbiome is the primary cause of these traits, the two can be told apart, and they sometimes appear independently from one another.

Today’s microbiome-phenotype determinism is grounded on the almost exclusive reliance on whole-microbiome empirical studies, together with the way in which the results from these studies are interpreted. Empirical studies grounded on microbiome-phenotype determinism frequently seek to understand how different phenotypic states correlate with microbiome differences. There are two main types of studies. Firstly, wide-population studies screening off the microbiome of healthy and unhealthy donors—mostly humans—to establish the differences in microbiome composition and/or abundance. An underlying assumption of these studies is that the microbiome composition must play a role in the different pathological states of the host, and thus the microbiome is interpreted as an agent of host disease. For example, several studies have shown how microbiome differences are associated to mental diseases, such as schizophrenia or dementia, and also in other types of diseases such as different types of cancer, diabetes or intestinal bowel disease (for a summary, see Madhogaria et al., 2022). Some studies of this type include Nguyen et al. (2019), Shen et al. (2018), Evans et al. (2017), Flowers et al. (2017), Coit et al. (2016). Secondly, studies with gnotobiotic model organisms, including studies with germ-free and/or artificially colonized animals. In these studies, it is tested how a germ-free animal develops, and how some of its normal physiological features are affected when reared free of germs, and/or when germs are inoculated later than normal in their lifespan. This last case includes inoculation of the microbiome from unhealthy donors, to detect whether the response would be the same in the germ-free organism than it was in the unhealthy donor. An underlying assumption is that these studies will reveal the causal role of the microbiome in the host phenotype. This assumption is specifically manifested in the way in which other factors and tools to study the microbiome effects on the host are ignored (for example, the role of ecological assembly or the network structure of the microbiome). Examples of research with gnotobiotic organisms include Kelly et al. (2016), Zheng et al. (2016), Shen et al. (2017), Hsiao et al. (2013), Neufeld et al. (2011).

In most contemporary studies applying any of these methods, the microbiome as a whole is taken as a primary source of information about a host trait (methodological microbiome-phenotype determinism), and/or the causal agent underlying some of its phenotypic traits (ontological microbiome-phenotype determinism). The second alternative was already criticized about one decade ago by Hanage (2014) and, more recently, by several other authors (Cani, 2018; Bourrat, 2018; Walter et al., 2020; Lynch et al., 2019; O’Malley & Parke, 2020). The main point in all this research is that the association of changes in microbiome composition with changes in host traits is far from showing that the former causes the latter, as some of the well-known and accepted conditions to establish causality in biomedicine are not fulfilled. I would however make the point slightly differently, for in my view the main problem with this type of research is not the attribution of causality to the microbiome, but the specific form that this causality is supposed to take and its association with the dangerous form of determinism denounced in this work.^5^ Concretely, the problem is that small changes in microbiome composition between hosts or within the same host are believed to play a primary causal role in the production of the trait. Yet, these microbiome differences are analysed at a taxonomic level (using e.g., taxonomic markers such as 16S rRNA, see Suárez & Boem, 2022), or, at best, by using tools such as proteomics or metabolomics that reveal the type of products that the microbiome produces. The problem, though, is that these types of analyses fail to go beyond this molecular lower-level; i.e., they do not try to understand whether the host-microbiome higher-level interactions themselves may be causally responsible of these changes (Sect. 4). The causal primacy of the microbiome in these studies, thus, is reflected as a causal primacy of the molecular, which may mask stronger causal effects such as those produced via the complex ecological, immunological or evolutionary interactions between the host and its microbiome. This complain is similar to Kaplan’s complain about the excessive reliance on genetic foundations in the Human Genome Project, in detriment of other contextual factors that may ultimately determine what the genetic produces. In this sense, ontological microbiome-phenotype determinism shares the same type of problems as ontological genetic determinism.

In a recent work that matches well with my criticisms here, Schneider (2023) denounces how microbiome studies rely on these types of molecular methods and ignore other available tools, such as those coming from our knowledge about ecological theory. Schneider contends that the microbiome is a an ecological community and thus the promise that we gain any knowledge of how the microbiome primarily causes host traits (ontological microbiome-phenotype determinism) or allows explanation and prediction of these traits (methodological microbiome-phenotype determinism) crucially depends on that knowledge.^6^ While I do not fully share Schneider’s ecological view of the microbiome, I think the main lesson one can draw from her work is that a methodological problem in contemporary microbiome science is how its reliance on molecular methods leads to ignoring alternative methodologies (mainly, those relying on ecological methods) that may play a fundamental role in investigating, predicting or explaining the role of the microbiome in producing traits with partial microbiome aetiologies. This form of methodological microbiome-phenotype determinism is thus a reality in contemporary microbiome science, and once that must be resisted.

Overall, microbiome-phenotype determinism, in both the methodological and the ontological form, singles out the genetic part of the microbiome as a privileged factor in predicting and manipulating host biology. In the next section, I analyse the main problems underlying genetic determinism in microbiome science.

## Problems of microbiome determinism

Understanding the limitations, perils and false hopes of the two types of determinism in microbiome science analysed in this work requires first understanding the nature of host-microbiome associations. Particularly, it requires understanding the complexities of the interactions, how these are built and maintained, how these can change during host development and evolution and, importantly, how these are stabilized and can be destabilized. This is because the whole question surrounding determinism is whether an attitude that privileges a methodology and an ontology grounded on the primacy of some parts over the rest of parts of the whole in explaining/predicting and controlling trait expression is coherent with our scientific knowledge of the relationship between the privileged parts and the other parts of the whole. In other words, do the parts deemed responsible for the primacy over the control *really* have that primacy? And, if they do so, do they acquire that primacy regardless of the context, or is their primacy just a mere causal influence when the context is favourable for that type of action? In the specific case of microbiome science, these two questions may be asked with regards to the host-microbiome determinism and microbiome-phenotype determinism.

A possible way of replying to these questions for host-microbiome determinism would consist in showing that it is empirically false that the host genetically controls microbiome assembly (ontological thesis). Rather, the microbiome is assembled de novo in each generation and the evidence suggests that this assembly depends on the host ecological opportunities, rather than on the host genetics, as many authors have shown (e.g. Moran & Sloan, 2015; Douglas & Werren, 2016; Skilling, 2016; Hurst , 2017; Bourrat & Griffiths, 2018; Stencel & Wloch-Salamon, 2018; Koskella & Bergelson, 2020; Roughgarden, 2023). Hence, the host genetics is not be best place to primarily study microbiome assembly (methodological thesis). Note that this does not deny that the host genotype may play *some* causal role in determining the microbiome assembly. It simply states that it does not play a primary role, and thus any of the methods I described in Sect. 3 as fostering host-microbiome determinism should be complemented with others studying other potential factors determining microbiome assembly. For example, studies on the ecology of the microbiome, or studies on how specific environmental factors may drive the acquisition of certain microbiome components, or the acquisition of specific microbiome functions (Boem & Suárez 2024).

However, while this argument could work for the specific case of host-microbiome determinism, I do not think it would be a fully convincing strategy to oppose microbiome-phenotype determinism. For while the critics would have a powerful empirical claim against the host genotype having a primary causal influence on microbiome assembly, this argument does not touch on the conceptual and empirical grounds of microbiome-phenotype determinism, though. There are two reasons for this. Firstly, conceptually speaking, rejecting that the host genotype plays a primary causal role in assembling the microbiome is consistent with the thesis that, if it did so, then the microbiome would be a primary causal factor in producing the host phenotype. To put it differently, it is consistent with stating that if the assembly of the whole microbiome, or the assembly of some microbes in the microbiome (e.g. the mitochondria in eukaryotic cells), were controlled by the host genotype (e.g., by controlling its mode of transmission), then the microbiome could control the expression of certain traits of its host (ontological thesis), and/or should be the primary source for investigating these traits (methodological thesis). Secondly, denying that the host genotype is the primary causal factor of the microbiome assembly leaves untouched the empirical question about the causal relationship between the microbiome and the host phenotype. This is because it may happen that the microbiome plays a primary role in determining the host phenotype even if the microbiome were environmentally acquired. All that is required is that the microbiome bears some genes that play a primary causal role in the production of some host traits (ontological thesis) and thus the microbiome would be the best source to predict or explain why the host bear those traits (methodological thesis). In short, the mode of acquisition of the microbiome is, to a certain extent, empirically irrelevant to explore the validity of microbiome-phenotype determinism. Therefore, I claim this not to be a good conceptual move against microbiome-phenotype determinism.

A more promising avenue to contest microbiome-phenotype determinism consists in pointing out the nuances of phenotype expression, as well as the specific role that the microbiome may play in that phenomenon. Concretely, I refer to three well-described biological phenomena that question microbiome-phenotype determinism: (1) epistasis plays a key, foundational role, in the type of synergies driving host-microbiome associations; (2) not every part of the microbiome can play a causal role for phenotypic expression, but only discrete traits which are frequently redundant; (3) host-microbiome systems exhibit a network structure, in which network properties play a more determinant role on what happens to the parts than the other way around. While these three phenomena are in the end interconnected, they can be analytically separated to facilitate the study of how each of them independently questions the validity of microbiome determinism in any of the two forms isolated in Sect. 3. Particularly, I argue that: (1) epistasis questions methodological microbiome-phenotype determinism; (2) microbiome redundance suggests the untenability of methodological and ontological host-microbiome determinism; (3) the network structure of host-microbiome systems challenges the possibility of ontological microbiome-phenotype determinism. Let us deal with these points in detail.

*Epistasis*. Epistasis refers to the non-additive interactions between genes—alleles—in different loci that result in an expressed phenotype which is different from the phenotype that would be expressed if each of the interacting genes would have acted separately. Evidence supporting the role of epistasis in determining several phenotypic effects is abundant, and so are the long term effects of epistatic interactions between genes (Phillips, 2008). Epistasis between the host and either the taxa or the functional traits of its microbiome is speculated to be a primary driver of host-microbiome interactions, and several theoretical and empirical studies have already proven this to be so (Bourrat, 2019; Lloyd & Wade, 2019). As a matter of fact, epistasis is hypothesised to underlie the main biological processes leading to host-microbiome evolution, including the potential for the evolution of mutualism and vertical transmission.

Epistasis poses a serious obstacle for the possibility of methodological microbiome-phenotype determinism due to the non-linearity of the interactions among genes at different loci, and the fact that this non-linearity eventually leads to emergent phenotypes, as consistently documented in the literature on emergence (Bedau, 1997; Suárez & Triviño, 2019, 2020; Wilson, 2016).^7^ Microbiome-phenotype methodological determinism works by assuming that the microbiome is the most adequate place to investigate, predict or explain host traits with partial microbiome aetiologies. Thus, it leads to research methodologically comparing the microbiome of hosts expressing the trait of interest *x*, and those not expressing it or expressing a different value of the trait—if the trait is quantitative. This hypothesis would be feasible if the interactions between the genes in the microbiome, or between the microbiome and the host, were merely additive. In that case, it would be possible to predict host states from microbiome states, and the likelihood that the microbiome primarily controls the expression of some traits would increase. In contrast with this, epistatic interactions are non-linear, both in the host-microbiome relationship and in the interrelationships between the different components of the microbiome. Therefore, the specific phenotypic state that the host expresses turns out to be unpredictable. This is important because it suggests that instead of interpreting the work on mGWAS or on gnotobiotic research as if it suggested that some differences in the microbiome may allow the prediction of differences in the expressed traits, it should be read the other way around: as if some differences *and/or analogies* in the expressed traits may allow the prediction of differences in the microbiome. That is, as if the differences or similarities in the microbiome were methodologically irrelevant to account for the differences in the expressed phenotype at the higher-level, because epistasis precisely suggests that the higher-level is a better predictor of what happens at the lower-level. Therefore, epistasis questions the possibility of any form of methodological microbiome-phenotype determinism insofar as it entails admitting that that the expression of the traits will depend on their global context, and thus the microbiome cannot be the most adequate place to investigate, predict or explain host traits with partial microbiome aetiologies.

*Discrete and (mostly) redundant traits*. Most host-microbiome interactions do not affect the microbial components in the same way that they affect the host species, generating a host-centric unit based on the non-symmetry of the relationships between the components (Schneider, 2021; Stencel, 2022; Suárez & Stencel, 2020). This non-symmetry manifests in the interaction between the host species and a pool of variable bacterial species that bear or express similar traits. This leads to a functional redundancy of the microbiome, in which the traits contributing towards the global functionality of the host-microbiome system will tend towards stabilization, regardless of the species bearing them.

While the fact that discrete traits play the causal role in host microbiome systems does not necessarily hamper the possibility of developing a coherent and empirically grounded form of microbiome-phenotype determinism, it poses a serious challenge to the promise of host-microbiome determinism, both in its ontological and methodological variants. Let me sketch the reasons why this is so. If the main association is between the host and a set of discrete traits borne by the bacterial taxa, then it seems implausible that the host genetics may have any primary role in determining microbiome assembly. On the one hand, it seems evolutionarily implausible that the host genetics has an evolutionary primacy in determining which bacterial taxa the host associates with, especially given that the biologically relevant host interaction is not with the taxa but with some of the traits borne by these taxa. At most, microbiome assembly may be associated to host genetics by a common cause, but this is a far cry from the tenets of host-microbiome determinism which requires the host genetics to be a primary cause of microbiome assembly. On the other hand, even if one may correctly assume that host-microbiome determinism could be reformulated such that the deterministic primacy runs from the host genetics to the functions or traits expressed by the microbiome (Greslehner, 2020), the paradox would now be to explain how some traits, mostly known to be involved in abundant horizontal gene transfer and able to be borne by several bacterial taxa may guide microbiome assembly due to their association with the host genetics. Rather, what seems to be happening is that selection within the host-microbiome system *after assembly and during the lifespan of the host* drives the microbiome towards an optimal state where the necessary traits become dominant and redundant (Suárez, 2020). In fact, the empirical evidence suggests that colonization depends both on the context of the host as well as on bacteria-to-bacteria interactions, rather than on host control over bacterial assembly (Jones et al., 2022). This is important because it should oblige biologists to investigate other potential determinants of microbiome assembly, including host behaviour and/or host environment, as well as the role of the immunological system of the host and the very ecology of the microbiome in determining microbiome composition. But, as discussed in Sect. 3, microbiome-phenotype determinism, unfortunately, often precludes these investigations.

*Network structure of the host-microbiome system*. A system is said to possess a network structure when the association between its parts generate an emergent regime of causation due to the appearance of constraints resulting from the interaction between the parts. A constraint is a material structure that results from the interaction between the parts and harnesses their interactions, so that the range of possible states that each of the parts occupies is smaller than the potential states that it would occupy if no constraint existed (Emmeche et al., 2000; Umerez & Mossio, 2013). Green (2018), drawing on Emmeche et al. (2000), grounds the importance of the network structure on the existence of boundary conditions, “conditions under which a given mathematical model or equation hold (e.g., by specifying a value interval for the possible solution)” (Green, 2018, p. 1001; see also Green, 2020). Boundary conditions ontologically define how the higher-level constrains the behaviour of the lower-level parts, as their range of behaviours is always restricted to those delimited by the mathematical model defining the boundary conditions—which, by definition, must be narrower than the possibilities offered by the laws governing the behaviour of the lower-level parts. Simultaneously, a system with a network structure generates the potential for the appearance of new structures and functions that would be unlikely if the system lacked such structure (Moreno & Suárez, 2020).^8^

Two important features of network structures are their recursion and their resistance to perturbations or robustness. By the latter I mean that a system with a network structure is often capable of remaining in the same state—i.e., realizing the same dynamics—despite a range of potential threads that alter or affect their component parts (Green, 2022; Moreno & Suárez, 2020). In short, what happens when a network is perturbed is that it gets driven out of equilibrium, but the dynamics of a network are such that a new stable equilibrium will be reached after a certain time (Wagner 2005, for an analysis in terms of the concept of “distributional robustness”). This feature concerns the specific form of physical realization in network systems, being generally taken to entail a form of ontological emergence (Wilson, 2016), manifested by the strong degree of causal autonomy of the higher-level (in the case of the host-microbiome association, the host-microbiome system as a whole).

Different authors have already emphasised the network structure of host-microbiome systems (Doolittle & Booth, 2017; Doolittle & Inkpen, 2018; Huitzil et al., 2018; Bapteste & Papale, 2021). It is important to note, although in passing, that the realization of a network structure—regardless of the type, which varies across systems—affects all dimensions of these systems, including their ecology, evolution, physiology, etc. (Suárez, 2020). An important feature of most systems realizing a network structure is that it will be both robust—it will maintain its global, network properties despite perturbations or alterations at the lower-level—and resilient—capable of recovering quickly after a perturbation that the system is not vulnerable to (see also Green & Batterman, 2017). These two features definitely rule out the possibility of ontological microbiome-phenotype determinism. In a network system, what happens to the parts is conditioned upon the type of constraints that emerge from their interactions. In this sense, no part can be privileged over the rest, isolated, or attributed a function over and above the properties of the whole—the network. This is because the network exhibits a characteristic dynamic and every element that enters the network will be harnessed so that the dynamic is maintained. The network dynamics results from the very interactions between the elements, and even if the host assumes the role of a central node within the network, which is something known to happen in host-microbiome systems (Suárez & Stencel, 2020), its behaviour cannot be reduced to the behaviour of the host. This also affects the microbiome and its potential for affecting the phenotypic expression of the host. It is true the microbiome may alter that expression, but only if it is able to bring the whole host-microbiome network towards a new dynamic. For this to happen, the changes in the microbiome need to be structural, such that the action of the host, or the action of other components of the microbiome, do not hamper the possible changes brought about by the former. In this context, the possibility that the microbiome primarily controls any host trait is ruled out. At most, alterations in the microbiome can bring about new dynamics to the host-microbiome system, provided the former does not resit these alterations. Importantly, these new dynamics will likely be temporal, as the host-microbiome network will tend towards a stable equilibrium. Even when these alterations are not temporal, the causality is not linearly produced, from the microbiome to the host. It is rather a network dynamic. Therefore, ontological microbiome-phenotype determinism in the form defined in this paper is untenable.

## Conclusion

This paper argued that, despite the promises that microbiome science could definitely debunk some deterministic hypotheses originally embedded in the Human Genome Project, an important part of contemporary research in microbiome science is grounded on deterministic theses. Concretely, I argued that some of the gene determinism underlying the Human Genome Project was expanded and became applicable to study the causal influences between symbiotic organisms. In microbiome science, determinism primarily permeated through the methods being used—mainly molecular and focusing on the microbiome species composition, rather than on other biological characteristics—and the approaches being neglected—for example, ecological approaches. Grounded on this, I distinguished two forms of determinism in contemporary microbiome science: host-microbiome determinism and microbiome-phenotype determinism. I showed that both forms of determinism presuppose the ideas of “genetic control” and “methodological primacy of the genetic” that permeated genomic determinism. Due to the nuances of microbiome science, I also showed that these approaches permeated in a distinctive way: namely, by reducing microbiome research to molecular methods, mainly gene-centred based methodologies, and ignoring other types of methodologies currently available, such as those based on ecological methods. Secondly, by relying on recent research on host-microbiome systems, I argued that these forms of determinism score poorly with our best ways of characterizing the relationships between microbiomes and their hosts. Concretely, I first argued that our current evidence suggests that the host genotype is not the main factor driving microbiome assembly, thus questioning host-microbiome determinism; second, I emphasized that the role of epistasis in shaping host-microbiome interactions, the fact that host-microbiome interactions do not affect the whole microbiome, or even the species composition of the microbiome, but rather microbial traits, and the network structure of the microbiome, jointly make questionable the main assumptions underlying microbiome-phenotype determinism and host-microbiome determinism.

I conclude by encouraging others to investigate other possible forms of microbiome determinisms I may have missed. In addition, I suggest, that more research needs to be conducted on how the microbiome may still be an important causal factor contributing to variation in the host phenotype, as well as how causality generally acts in the microbiome, beyond the constraints imposed by any (gene) deterministic programme. Finally, I encourage researchers to address other factor than transcend the genetic level when uncovering the primary causes of phenotypic traits in order to avoid that microbiome research repeats the same ontological and epistemological mistakes that were on the basis of the Human Genome Project.

## Footnotes

1 For an excellent clarification of the distinction between genetic reductionism, genocentrism and genetic determinism, see Sarkar (1998, pp. 10–13), Kitcher (2001), Rosoff and Rosenberg (2006).

2 Additionally, the paper is not about the big claims concerning whether—and how—the microbiome affects what it means to be human (Nieves Delgado & Baedke, 2021; Parke, 2021; Rees et al., 2018). This would be a question about essentialism vs. non-essentialism, whereas the question I aim to address concerns determinism. Finally, even if the paper relies on a variety of empirical studies grounded on the use of different models—including different sequencing technologies and different model organisms—this paper is not about whether these models justify some of the potential conclusions drawn about the microbiome *in general* (Douglas, 2018; Suárez & Boem, 2022; Walter et al., 2020), but rather whether these models justify deterministic conclusions.

3 See next section for the evidence of how these two types of determinism are introduced in the contemporary literature.

4 Note that the distinction between host-microbiome determinism and microbiome-phenotype determinism is analytic, to better understand what’s at stake in different studies. However, most host-microbiome deterministic studies motivate the research based on the phenotypic effects that the microbiome has on its host. This does not mean that host-microbiome determinism entails microbiome-phenotype determinism, as the former is compatible with a “causal influence” perspective on the microbiome-phenotype association. Similarly, even if most microbiome-phenotype deterministic studies also presuppose a form of host-microbiome deterministic hypothesis as well, this is not always the case, nor does it need to be the case.

5 I already made clear in Sect. 1 that this paper is not about causality, and explained the reasons why, so I will not repeat them here.

6 And, crucially and importantly, ecological knowledge would be higher-level and holistic, rather than solely molecular and deterministic.

7 It specifically leads to epistemological or Bedau’s *weak* emergence, manifested for the lack of predictability due to the way in which very small context changes lead to severe changes in the effects produced by the factor. In this case, due to the drastic changes in microbiome behaviour due to minimal changes in the host (e.g., a small change in its health status). Note that epistasis does not necessarily lead to any form of metaphysical emergence, and thus it would not suppose any obstacle to ontological microbiome-phenotype determinism.

8 I am conscious that this opens up the possibility that causation in network systems works downwardly, instead of upwardly, as several authors have recently suggested (Green, 2018, 2020; Green & Battermann, 2017, 2020; Woodward, 2020) and others have speculatively applied to host-microbiome systems (Suárez & Triviño, 2019; Ronai et al., 2020). I will leave the exploration of such possibility for another work.

## References

[CR3] Bedau MA (1997). Weak emergence. Philosophical Perspectives.

[CR4] Baedke J (2019). O organism, where art thou? Old and new challenges for organism-centered biology. Journal of the History of Biology.

[CR5] Bapteste E, Papale F (2021). Modeling the evolution of interconnected processes: It is the song and the singers. BioEssays.

[CR6] Blekhman R, Goodrich JK, Huang K, Sun Q, Bukowski R, Bell JT, Spector TD, Keinan A, Ley RE, Gevers D, Clark AG (2018). Host genetic variation impacts microbiome compositions across human body sites. Genome Biology.

[CR7] Boem, F., & Suárez, J. (2024). Pistemic misalignments in microbiome research. *BioEssays*. 10.1002/bies.202300220 38403799

[CR8] Bonder MJ, Kurilshikov A, Tigchelaar EF, Mujagic Z, Imhann F, Vila AV, Deelen P, Vatanen T, Schirmer M, Smeekens SP, Zhernakova DV (2016). The effect of host genetics on the gut microbiome. Nature Genetics.

[CR9] Bourrat P (2018). Have causal claims about the gut microbiome been over-hyped?. BioEssays.

[CR10] Bourrat P (2019). Evolutionary transitions in heritability and individuality. Theory in Biosciences.

[CR11] Bourrat P, Griffiths P (2018). Multispecies individuals. History and Philosophy of the Life Sciences.

[CR12] Cani PD (2018). Human gut microbiome: Hopes, threats and promises. Gut.

[CR14] Chiu L, Gilbert SF (2015). The birth of the holobiont: Multi-species birthing through mutual scaffolding and niche construction. Biosemiotics.

[CR15] Coit P, Mumcu G, Ture-Ozdemir F, Unal AU, Alpar U, Bostanci N, Ergun T, Direskeneli H, Sawalha AH (2016). Sequencing of 16S rRNA reveals a distinct salivary microbiome signature in Behçet's disease. Clinical Immunology.

[CR16] Davenport ER, Cusanovich DA, Michelini K, Barreiro LB, Ober C, Gilad Y (2015). Genome-wide association studies of the human gut microbiota. PLoS ONE.

[CR17] Doolittle WF, Booth A (2017). It’s the song, not the singer: An exploration of holobiosis and evolutionary theory. Biology & Philosophy.

[CR18] Doolittle WF, Inkpen SA (2018). Processes and patterns of interaction as units of selection: An introduction to ITSNTS thinking. PNAS.

[CR19] Douglas AE (2018). Which experimental systems should we use for human microbiome science?. PLOS Biology.

[CR21] Douglas AE, Werren JH (2016). Holes in the hologenome: Why Host-microbe symbioses are not holobionts. Mbio.

[CR22] Dove NC, Veach AM, Muchero W, Wahl T, Stegen JC, Schadt CW, Cregger MA (2021). Assembly of the Populus microbiome is temporally dynamic and determined by selective and stochastic factors. mSphere.

[CR23] Dubois M, Guaspare C, Louvel S (2018). De la génétique à l’épigénétique: Une révolution ‘post-génomique’ à l’usage des sociologues. Revue Française De La Sociologie.

[CR24] Dupras C, Saulnier KM, Joly Y (2019). Epigenetics, ethics, law and society: A multidisciplinary review of descriptive, instrumental, dialectical and reflexive analyses. Social Studies of Science.

[CR25] Dupré J (2003). Human nature and the Limits of Science.

[CR26] Dupré J (2010). The polygenomic organism. The Sociological Review.

[CR27] Dupré J (2021). Causally powerful processes. Synthese.

[CR28] Ellison GTH, De Wet T, Trevathan W (2018). Biological determinism. The International Encyclopedia of Biological Anthropology.

[CR29] Emmeche C, Køppe S, Stjernfelt F, Andersen PB, Emmeche C, Finnermann NO, Christiansen PV (2000). Levels, emergence, and three versions of downward causation. Downward causation. Minds, bodies and matter.

[CR30] Evans SJ, Bassis CM, Hein R (2017). The gut microbiome composition associates with bipolar disorder and illness severity. Journal of Psychiatric Research.

[CR31] Falony G, Joossens M, Vieira-Silva S (2016). Population-level analysis of gut microbiome variation. Science.

[CR32] Flowers SA, Evans SJ, Wards KM, McInnis MG, Ellingrod VL (2017). Interaction between atypical antipsychotics and the gut microbiome in a bipolar disease cohort. Pharmacotherapy.

[CR33] Fox Keller E (2000). The century of the gene.

[CR34] Furman O, Shenhav L, Sasson G, Kokou F, Honig H, Jacoby S, Hertz T, Cordero OX, Halperin E, Mizrahi I (2020). Stochasticity constrained by deterministic effects of diet and age drive rumen microbiome assembly dynamics. Nature Communications.

[CR35] Gannett, L. (2019). The human genome project. In E. N. Zalta (Ed.), *The Stanford encyclopedia of philosophy*. https://plato.stanford.edu/entries/human-genome/. Last Access 12 Mar 2022.

[CR36] Gayon J, Kupiec J-J, Gandrillon O, Morange M, Silberstein M (2009). Déterminisme génétique, déterminisme bernardien, déterminisme laplacien. Le hasard au coeur de la cellule.

[CR37] Gilbert SF, Sapp J, Tauber AI (2012). A symbiotic view of life: We have never been individuals. The Quarterly Review of Biology.

[CR38] Goodrich JK, Waters JL, Poole AC, Sutter JL, Koren O, Blekhman R, Beaumont M, Van Treuren W, Knight R, Bell JT, Spector TD (2014). Human genetics shape the gut microbiome. Cell.

[CR39] Goodrich JK, Di Rienzi SC, Poole AC, Koren O, Walters WA, Caporaso JG, Knight R, Ley RE (2014). Conducting a microbiome study. Cell.

[CR40] Goodrich J, Davenport ER, Waters JL, Clark AG, Ley RE (2016). Cross-species comparisons of host genetic associations with the microbiome. Science.

[CR41] Green S (2018). Scale-dependency and downward causation in biology. Philosophy of Science.

[CR42] Green S, Brooks DS, DiFrisco J, Wimsatt WC (2020). Cancer beyond genetics: On the practical implications of downward causation. Biological levels: Composition, scale and evolution in complex systems.

[CR43] Green, S. (2022) Philosophy of systems and synthetic biology. In E. N. Zalta (Ed.). *The Stanford encyclopedia of philosophy*. https://plato.stanford.edu/entries/systems-synthetic-biology/. Last Access 22 May 2022.

[CR44] Green S, Batterman R (2017). Biology meets physics: Reductionism and multi-scale modelling of morphogenesis. Studies in History and Philosophy of Science Part C.

[CR45] Green S, Battermann R, Boosholz J, Gabriel M (2020). Making sense of top-down causation: Universality and Functional Equivalence in Physics and Biology. Top-down causation and emergence.

[CR46] Greslehner G (2020). Not by structures alone: Can the immune system recognize microbial functions?. Studies in History and Philosophy of Science Part c: Studies in History and Philosophy of Biological and Biomedical Sciences.

[CR47] Griffiths P, Stotz K (2013). Genetics and philosophy.

[CR48] Grunstra NDS, Zachos FE, Herdina AN, Fischer B, Pavličev M, Mitteroecker P (2019). Humans as inverted bats: A comparative approach to the obstetric conundrum. American Journal of Human Biology.

[CR49] Hanage WP (2014). Microbiology: Microbiome science needs a healthy dose of scepticism. Nature.

[CR50] Hoefer, C. (2016) Causal determinism. In E. N. Zalta (Ed.). *The Stanford encyclopedia of philosophy*. https://plato.stanford.edu/entries/determinism-causal/. Last Access 12 Mar 2022.

[CR51] Hsiao EY, McBride SW, Hsien S, Sharon G, Hyde ER, McCue T, Codelli JA, Chow J, Reisman SE, Petrosino JF, Patterson PH (2013). Microbiota modulate behavioral and physiological abnormalities associated with neurodevelopmental disorders. Cell.

[CR52] Hua X, Goedert JJ, Landi MT, Shi J (2016). Identifying host genetic variants associated with microbiome composition by testing multiple beta diversity matrices. Human Heredity.

[CR53] Huitzil S, Sandoval-Motta S, Frank A, Aldana M (2018). Modeling the role of the microbiome in evolution. Frontiers in Physiology.

[CR54] Hurst GDD (2017). Extended genomes: Symbiosis and evolution. Interface Focus.

[CR55] Igartua C, Davenport ER, Gilad Y, Nicolae DL, Pinto J, Ober C (2017). Host genetic variation in mucosal immunity pathways influences the upper airway microbiome. Microbiome.

[CR57] Jones EW, Carlson JM, Sivak DA, Ludington WB (2022). Stochastic microbiome assembly depends on context. PNAS.

[CR58] Kaplan JM (2000). The limits and lies of human genetics research: Dangers for social policy.

[CR129] Kelly, J. R., Borre. Y., O' Brien, C., Patterson, E., El Aidy, S., Deane, J., Kennedy, P. J., Beers, S., Scott, K., Moloney, G., Hoban, A. E., Scott, L., Fitzgerald, P., Ross, P., Stanton, C., Clarke, G., Cryan, J. F., & Dinan, T. G. (2016). Transferring the blues: Depression-associated gut microbiota induces neurobehavioural changes in the rat. *Journal of Psychiatric Research,**82,* 109-118. 10.1016/j.jpsychires.2016.07.019 27491067

[CR59] Koskella B, Bergelson J (2020). The study of host–microbiome (co)evolution across levels of selection. Philosophical transactions of the Royal Society.

[CR60] Kitcher P (2001). Science, truth, and democracy.

[CR61] Le Roy CI, Beaumont M, Jackson MA, Steves CJ, Spector TD, Bell JT (2018). Heritable components of the human fecal microbiome are associated with visceral fat. Gut Microbes.

[CR62] Lloyd EA, Wade MJ (2019). Criteria for holobionts from community genetics. Biological Theory.

[CR63] Lynch JB, Hsiao EY (2019). Microbiomes as sources of emergent host phenotypes. Science.

[CR64] Lynch KE, Parke EC, O’Malley MA (2019). How causal are microbiomes? A comparison with the *Helicobacter pylori* explanation of ulcers. Biology and Philosophy.

[CR128] Madhogaria B., Bhowmik P., & Kundu A. (2022). Correlation between human gut microbiome and diseases. *Infectious Medicine**1*(3), 180-191. (Beijing) 10.1016/j.imj.2022.08.004 PMC10699709 38077626

[CR65] McFall-Ngai M, Hadfield MG, Bosch TC, Carey HV, Domazet-Lošo T, Douglas AE, Dubilier N, Eberl G, Fukami T, Gilbert SF, Hentschel U (2013). Animals in a bacterial world, a new imperative for the life sciences. PNAS.

[CR66] Meloni M, Testa G (2014). Scrutinizing the epigenetics revolution. BioSocieties.

[CR67] Merlin F, Roboli-Sasco L (2021). Inheritance as evolved and evolving physiological processes. Acta Biotheoretica.

[CR68] Montévil M, Longo G, Soto A, Gaudin T, Lacroix D, Maurel M-C, Pomerol J-C (2018). From the century of the gene to that of the organism: Introduction to new theoretical perspectives. Life sciences, information sciences.

[CR69] Moran NA, Sloan DB (2015). The hologenome concept: Helpful or hollow?. PLOS Biology.

[CR70] Moreno A, Suárez J, Gonzalez WJ (2020). Plurality of explanatory strategies in biology: Mechanisms and networks. Methodological prospects for scientific research: From pragmatism to pluralism.

[CR71] Müller T, Placek T (2018). Defining Determinism. The British Journal for the Philosophy of Science.

[CR72] Nagpal J, Cryan JF (2021). Host genetics, the microbiome and behaviour—a ‘Holobiont’ perspective. Cell Research.

[CR73] Neufeld KM, Kang N, Bienenstock J, Foster JA (2011). Reduced anxiety-like behavior and central neurochemical change in germ-free mice. Neurogastroenterology and Motility.

[CR74] Nguyen TT, Kosciolek T, Maldonado Y, Daly RE, Martin AS, McDonald D, Knight R, Jeste DV (2019). Differences in gut microbiome composition between persons with chronic schizophrenia and healthy comparison subjects. Schizophrenia Research.

[CR75] Nieves Delgado A, Baedke J (2021). Does the human microbiome tell us something about race?. Humanities and social Sciences Communications.

[CR76] O’Malley, M. A., & Parke, E. C. (2020). Philosophy of microbiology. In E. N. Zalta (Ed.), *The Stanford encyclopedia of philosophy*. https://plato.stanford.edu/archives/fall2020/entries/microbiology/. Last access 21 May 2022.

[CR77] Parke EC (2021). Trivial, interesting, or overselling? The microbiome and ‘what it means to be human’. BioScience.

[CR78] Phillips P (2008). Epistasis—the essential role of gene interactions in the structure and evolution of genetic systems. Nature Reviews Genetics.

[CR79] Rawls JF, Mahowals MA, Ley RE, Gordon JI (2006). Reciprocal gut microbiota transplants from zebrafish and mice to germ-free recipients reveal host habitat selection. Cell.

[CR80] Rees T, Bosch T, Douglas AE (2018). How the microbiome challenges our concept of self. PLOS Biology.

[CR81] Richardson S, Stevens H (2015). Postgenomics: Perspectives on biology after the genome.

[CR82] Ronai I, Greslehner GP, Boem F, Carlisle J, Stencel A, Suárez J, Bayir S, Bretting W, Formosinho J, Guerrero AC, Morgan WH (2020). “Microbiota, symbiosis and individuality summer school” meeting report. Microbiome.

[CR83] Roughgarden J (2023). Holobiont evolution: Population theory for the hologenome. The American Naturalist.

[CR84] Rosoff PM, Rosenberg A (2006). How Darwinian reductionism refutes genetic determinism. Studies in History and Philosophy of Science Part c: Studies in History and Philosophy of Biological and Biomedical Sciences.

[CR85] Rothschild D, Weissbrod O, Barkan E, Kurilshikov A, Korem T, Zeevi D, Costea PI, Godneva A, Kalka IN, Bar N, Shilo S (2018). Environment dominates over host genetics in shaping human gut microbiota. Nature.

[CR86] Roubertoux PL, Carlier M (2011). Good use and misuse of “genetic determinism”. Journal of Physiology-Paris.

[CR87] Rühlemann MC, Degenhardt F, Thingholm LB, Wang J, Skiecevičienė J, Rausch P, Hov JR, Lieb W, Karlsen TH, Laudes M, Baines JF (2018). Application of the distance-based F test in an mgwas investigating beta diversity of intestinal microbiota identifies variants in slc9a8 (nhe8) and 3 other loci. Gut Microbes.

[CR88] Sahu M, Prasuna JG (2016). Twin studies: A unique epidemiological tool. Indian J Community Med..

[CR89] Sandoval-Motta S, Aldana M, Martínez-Romero E, Frank A (2017). The human microbiome and the missing heritability problem. Frontiers in Genetics.

[CR90] Santaló J, Camprubí C, Blanco J (2018). Epigenetics: Hope against genetic determinism. Epigenetics and assisted reproduction. An introductory guide.

[CR91] Sarkar S (1998). Genetics and reductionism.

[CR92] Schneider T (2021). The holobiont self: Understanding immunity in context. HPLS.

[CR93] Schneider T (2023). The microbiome function in a host organism: A medical puzzle or an essential ecological environment?. Biological Theory.

[CR94] Shen K, Liu L, Ji H-F (2017). Alzheimer’s disease histological and behavioral manifestations in transgenic mice correlate with specific gut microbiome state. Journal of Alzheimer's Disease.

[CR95] Shen Y, Xu J, Li Z, Huang Y, Yuan Y, Wang J, Zhang M, Hu S, Liang Y (2018). Analysis of gut microbiota diversity and auxiliary diagnosis as a biomarker in patients with schizophrenia: A cross-sectional study. Schizophrenia Research.

[CR97] Skillings D (2016). Holobionts and the ecology of organisms: Multi-species communities or integrated individuals?. Biology and Philosophy.

[CR100] Stencel A (2022). Why the evolution of heritable symbiosis neither enhances nor diminishes the fitness of a symbiont. Philosophy, Theory, and Practice in Biology.

[CR101] Stencel A, Proszewska A (2017). How research on microbiomes is changing biology: A discussion of the concept of the organism. Foundations of Science.

[CR102] Stencel A, Wloch-Salamon DM (2018). Some theoretical insights into the hologenome theory of evolution and the role of microbes in speciation. Theory in Biosciences.

[CR103] Suárez J (2018). The importance of symbiosis in philosophy of biology: An analysis of the current debate on biological individuality and its historical roots. Symbiosis.

[CR104] Suárez J (2020). The stability of traits conception of the hologenome: An evolutionary account of holobiont individuality. HPLS.

[CR105] Suárez J, Boem F (2022). Technology-driven surrogates and the perils of epistemic misalignment: An analysis in contemporary microbiome science. Synthese.

[CR106] Suárez J, Lloyd EA (2023). Units of selection.

[CR107] Suárez J, Stencel A (2020). A part-dependent account of biological individuality: Why holobionts are individuals and ecosystems simultaneously. Biological Reviews of the Cambridge Philosophical Society.

[CR108] Suárez J, Triviño V (2019). A metaphysical approach to holobiont individuality: Holobionts as emergent individuals. Quaderns De Filosofia.

[CR109] Suárez J, Triviño V (2020). What is a hologenomic adaptation? Emergent individuality and inter-identity in multispecies systems. Frontiers in Psychology.

[CR110] Tam V, Patel N, Turcotte M, Bossé Y, Paré G, Meyre D (2019). Benefits and limitations of genome-wide association studies. Nature Reviews Genetics.

[CR111] Tims S, Derom C, Jonkers DM, Vlietinck R, Saris WH, Kleerebezem M, De Vos WM, Zoetendal EG (2013). Microbiota conservation and BMI signatures in adult monozygotic twins. ISME Journal.

[CR112] Turnbaugh P, Ley R, Hamady M, Fraser-Liggett CM, Knight R, Gordon JI (2007). The human microbiome project. Nature.

[CR113] Turnbaugh PJ, Hamady M, Yatsunenko T, Cantarel BL, Duncan A, Ley RE, Sogin ML, Jones WJ, Roe BA, Affourtit JP, Egholm M (2009). A core gut microbiome in obese and lean twins. Nature.

[CR114] Turpin W, Espin-Garcia O, Xu W, Silverberg MS, Kevans D, Smith MI, Guttman DS, Griffiths A, Panaccione R, Otley A, Xu L (2016). Association of host genome with intestinal microbial composition in a large healthy cohort. Nature Genetics.

[CR115] Umerez J, Mossio M, Dubitzky W, Wolkenhauer O, Cho K, Yokota H (2013). Constraint. Encyclopedia of systems biology.

[CR116] Veigl SJ, Suárez J, Stencel A (2022). Rethinking hereditary relations: the reconstitutor as the evolutionary unit of heredity. Synthese.

[CR117] Vuong HE, Yano JM, Fung TC, Hsiao EY (2017). The microbiome and host behavior. Annual Review of Neuroscience.

[CR118] Waggoner MR, Uller T (2015). Epigenetic determinism in science and society. New Genetics and Society.

[CR127] Wagner, A. (2005). Distributed robustness versus redundancy as causes of mutational robustness. *BioEssays 27*(2), 176-188. 10.1002/bies.20170 15666345

[CR119] Walter J, Armet AM, Finlay BB, Shanahan F (2020). Establishing or exaggerating causality for the gut microbiome: Lessons from human microbiota-associated rodents. Cell.

[CR120] Wang J, Thingholm LB, Skiecevičienė J, Rausch P, Kummen M, Hov JR, Degenhardt F, Heinsen FA, Rühlemann MC, Szymczak S, Holm K (2016). Genome-wide association analysis identifies variation in vitamin D receptor and other host factors influencing the gut microbiota. Nature Genetics.

[CR121] Weissbrod O, Rothschild D, Barkan E, Segal E (2018). Host genetics and microbiome associations through the lens of genome wide association studies. Current Opinion in Microbiology.

[CR122] Wilson J, Bigaj T, Wüthrich C (2016). Metaphysical emergence: Weak and strong. Metaphysics in contemporary physics.

[CR123] Woodward J, Boosholz J, Gabriel M (2020). Downward causation defended. Top-down causation and emergence.

[CR124] Xie H, Guo R, Zhong H, Feng Q, Lan Z, Qin B, Ward KJ, Jackson MA, Xia Y, Chen X, Chen B (2016). Shotgun metagenomics of 250 adult twins reveals genetic and environmental impacts on the gut microbiome. Cell Systems.

[CR125] Yatsunenko T, Rey FE, Manary MJ, Trehan I, Dominguez-Bello MG, Contreras M, Magris M, Hidalgo G, Baldassano RN, Anokhin AP, Heath AC (2012). Human gut microbiome viewed across age and geography. Nature.

[CR126] Zheng P, Zeng B, Zhou C, Liu M, Fang Z, Xu X, Zeng L, Chen J, Fan S, Du X, Zhang X (2016). Gut microbiome remodeling induces depressive-like behaviors through a pathway mediated by the host’s metabolism. Molecular Psychiatry.

